# The Medical Congress at Budapest

**Published:** 1908-06-06

**Authors:** 


### THE INTERNATIONAL MEDICAL CONGRESS AT BUDAPEST.

A meeting of the National Committee for Great Britain and Ireland of the sixteenth International Congress of Medicine was held at the rooms of the Medical Society of London on May 28, 1908, Dr. F. W. Pavy, F.R.S., in the chair. The National Committee was reconstituted by incorporating twenty-two new members with the former Committee. The officers of the new Committee are Dr. Pavy, F.R.S., President, and Mr. D'Arcy Power and Dr. Clive Riviere Hon. Secretaries. Letters were read from the Secretary General of the Congress stating that it would be held at Budapest, August 29 to September 4, 1909, under the patronage of his Imperial and Apostolic Royal Majesty Francis Joseph I. It was further reported that certain changes had been made in the method of obtaining scientific communications; no members or societies had been invited directly to contribute, but all papers and discussions had been left to the initiative of individuals. Under these circumstances the National Committee felt that it was necessary to urge those who proposed to read papers to send in the titles to the General Secretary (Professor Emil Grósz, Esterhazy-Utcza 7, Budapest, viii.) as soon as possible, because it is very desirable that the medical profession in the United Kingdom be adequately represented at the Congress. The work of the Congress is divided into twenty-one sections, of which the sixteenth section—otology—is to be held simultaneously with the eighth International Congress of Otology. The remaining sections are anatomy and embryology, physiology, general and experimental pathology, micro-biology (bacteriology) and pathological anatomy, therapeutics (which includes pharmacology, physical therapeutics, and balneology), internal medicine, surgery, obstetrics and gynæcology, ophthalmology, diseases of children, diseases of the nervous system, psychiatrics, dermatology and venereal diseases, diseases of the urinary tract, laryngology, otology, stomatology, hygiene and prophylactic medicine, medical jurisprudence, military and naval hygiene, tropical diseases. There will probably be the usual reduction in railway fares for those who attend the Congress, but no details are as yet forthcoming. Should a sufficient number of members from Great Britain and Ireland signify their intention of attending, it may be possible to obtain a special through train from Boulogne to Budapest. The cost of membership is 25 crowns, which is approximately £1 sterling for each member, and half that amount for members' wives and daughters. The money can be remitted by post-office money order payable to Professor Dr. Elischer, Treasurer of the Congress, at the current rate of exchange.

